# Replacing P-values with frequentist posterior probabilities of replication—When possible parameter values must have uniform marginal prior probabilities

**DOI:** 10.1371/journal.pone.0212302

**Published:** 2019-02-27

**Authors:** Huw Llewelyn

**Affiliations:** Department of Mathematics, Aberystwyth University, Penglais, Aberystwyth, United Kingdom; University of California Los Angeles, UNITED STATES

## Abstract

The prior probabilities of true outcomes for scientific replication have to be uniform by definition. This is because for replication, a study’s observations are regarded as samples taken from the set of possible outcomes of an ideally large continuation of that study. (The sampling is not done directly from some source population.) Therefore, each possible outcome is based on the same ideally large number of observations so that all possible outcomes for that study have the same prior probability. The calculation methods were demonstrated on a spreadsheet with simulated data on the distribution of people with an imaginary genetic marker. Binomial distributions are used to illustrate the concepts to avoid the effects of potentially misleading assumptions. Uniform prior probabilities allow a frequentist posterior probability distribution of a study result’s replication to be calculated conditional solely on the study’s observations. However, they can be combined with prior data or Bayesian prior distributions. If the probability distributions are symmetrical then the frequentist posterior probability of a true result that is equal to or more extreme than a null hypothesis will be the same as the one-sided P-value. This is an idealistic probability of replication within a specified range based on an assumption of perfect study method reproducibility. It can be used to estimate a realistic probability of replication by taking into account the probability of non-reproducible methods or subjects. A probability of replication will be lower if the subsequent outcome is a narrower range corresponding to a specified statistical significance, this being a more severe test. The frequentist posterior probability of replication may be easier than the P-value for non-statisticians to understand and to interpret.

## 1. Introduction

There is currently a crisis of confidence in the results of research in the medical, biological and social sciences because a higher than expected proportion of results are failing to be replicated [1]. Ioannides had already suggested some years ago that most scientific findings were probably false, partly because statistical methods were not being applied or interpreted properly [2]. Many scientists and their students find statistical principles difficult. Perhaps this is due to a missing link that also causes controversy amongst statisticians about how probability theory should be used when interpreting scientific data. The natural tendency of a scientist is to wish to estimate the probability that the outcome of a perfect version of a study based on an unlimited number of observations will fall within a specified range that includes the original study result, thus replicating it. It is widely assumed that this cannot be done based on a study’s data alone because the prior probabilities of such outcomes are unknown (e.g. in populations of people) and can only be guessed at. It is well recognised however that if the prior probabilities of possible true values during random sampling are assumed to be uniform, then according to Bayes’ rule, the probability of the null hypothesis or something more extreme will equal the one-sided P-value [3, 4, 5, 6].

Bayesian statisticians maintain that it is legitimate to specify a personal view of the prior probability distribution of possible true outcomes and then calculating a posterior probability of the true result falling into some range. Frequentist statisticians disagree and restrict themselves to calculating the likelihood of getting the observed data conditional on one or more possible true parameter values and not attempting to calculate the probabilities of true outcomes. Attempts have been made to calculate posterior probabilities by avoiding an explicitly Bayesian approach. For example, Colquhoun has emphasised that the probability of being wrong about some discovery is much higher than the P value and that this should be explored by using different simulations [7]. However, he did not address directly the probability of replicating a result. Kileen suggested calculating a probability of replicating a difference between an experimental result and control in the same direction when a study is repeated, based on the original P value alone [8]. For example, if the one-sided P value was 0.025 then the P-rep would be about (1+(0.025/(1−0.025))^2/3^)−1 = 0.92 [8, 9]. However, this result, which is based on a number of disputed assumptions [9], would be inconsistent with the probability of 0.975 of the null hypothesis or something more extreme by assuming a uniform or weak distribution for all possible hypothetical outcomes and applying a Bayesian calculation [3, 4, 5, 6]. Also there is no provision in Kileen’s P-rep approach for incorporating the probable effect of other data or methodological irregularities into the probability of replicating a result (e.g. P-hacking or ‘cherry picking’). The Open Science Collaboration estimated the probability of replication empirically by observing how frequently a number of studies would be replicated by resulting in a two-sided P-value of at least 0.05 [10]. This is a more severe standard than simply expecting a result to be less extreme than a null hypothesis.

This paper will show that in the case of replication, the possible outcomes to be predicted are those of a perfect study based on a large but a shared single number of observations. The possible outcomes are thus equally probable. This might be the missing link that could connect more securely the way that statisticians and scientists think. It was on this basis of a uniform prior probability distribution that Bayes [11] calculated the probability of a true proportion falling within any specified range during random sampling by applying the binomial distribution. In order to do this he appeared to use Bayes’ rule and to assume uniform prior probabilities (but did not state either explicitly). I shall try to show that Bayes was correct to think that during random sampling the postulated true values can be modelled using equal marginal prior probabilities. This may make it possible to apply Bayes’ rule in a way that makes statistical inference easier to understand by non-statisticians, an issue that is of concern at present [12] and which may be contributing to the replication crisis.

## 2. Methods and probability notation

### 2.1. The idea of replication

Scientists often wish to know what would probably happen if a study with a limited number of observations could continue until there were a very larger number of observations. The continuation of the study may have to be done by someone else, so a subsequent study could be regarded as an attempt to validate the initial study’s result. In principle, if a large group of studies had the same probability of 0.975 of replication within some specified range, and if such studies were continued until there were a large number of observations, then 97.5% of their results should fall within the specified range. If this were the case, the initial study result would have been replicated in 97.5% of cases. However, there has been uncertainty and disagreement in the scientific and statistical communities about how a probability of replication should be estimated.

**Fig 1:**
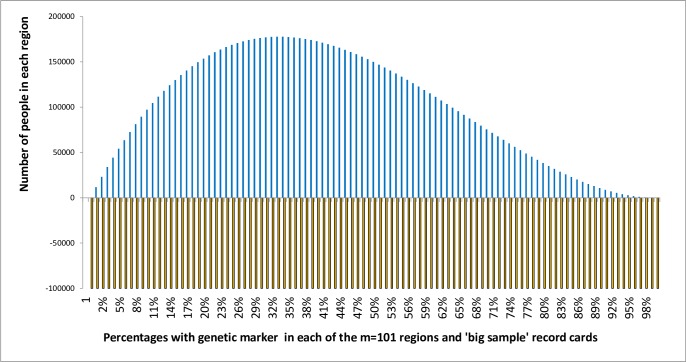
Number of people in each region (represented by the heights of the columns on the vertical axis) with each regional percentage possessing the genetic marker being represented on the horizontal axis and the equal number of random selection record cards represented by the rectangular distribution below the baseline.

The method used here to try to clarify the nature of replication is illustrated with hypothetical data, the calculations being made using an Excel spreadsheet that is freely available (see URL: https://osf.io/3utkj/?view_only=e0cc5791ed9b4a0899424a20e4611ccf, DOI 10.17605/OSF.IO/3UTKJ [13]). The example is based on people with a hypothetical genetic marker in a hypothetical population. The reader is asked to imagine 101 different hypothetical regions where the proportion of people with the genetic marker range from 0% to 100% and that these have been arranged in ascending order. In addition to this, the particular proportion with numbers of people in each group vary, the size of the population of the region with the genetic marker being shown by the height of the skewed distribution columns above the baseline in Fig 1.

Imagine that a scientist is given 101 boxes (each labelled ‘population box’) containing record cards for every individual in each of the 101 regions, recording whether the individual had the genetic marker or not. The scientist is not allowed to examine every population card in the box but allowed to select one at random, examine it, return it to the box and then select a card again and again at random from the box. Before returning each population card, its genetic marker status is written on another sample card, which is placed in another box labelled the ‘large sample box’. A large group of assistants make an ideally large 100,000 random selections with return from each of the 101 population boxes, thus creating 101 new large sample boxes, each containing exactly 100,000 randomly selected cards in each box. These are represented by the rectangular shaped distribution below the baseline in Fig 1. This means that the proportion *Θ*_*i*_ of cards with ‘genetic marker present’ written on them in each large sample box would be the same as the proportion *Θ*_*i*_ in each corresponding population box from which its cards had been selected. Note that the number of cards in the population box varies greatly (as indicated by the distribution in Fig 1) but these different population sizes do not affect the proportions in each large sample box at all. Each large sample box therefore contains a possible result of an idealised study based on a very large number of selections and results, each box containing the same number (e.g. of 100,000 cards) based on the observed result of a random selection.

A ‘large sample mystery box’ is placed in front of the scientist who is asked to guess the proportion of cards with ‘genetic marker present’ written on them in that box. This has to be done with a small number of random selections (e.g. 99). This would represent a practical study (unlike the ideal but impracticably large study of 100,000 observations). The task of the practical study of small random samples (e.g. 99) is to estimate the probability that the observed proportion of cards with ‘genetic marker present’ written on them had come from each of the 101 large sample boxes. The example calculations were performed in Microsoft Excel and are freely available to the reader together with all other calculations [13].

### 2.2. A comparison between sampling from the large sample boxes and the population boxes

If the scientist sampled from the population mystery box, she or he would be conducting an epidemiological study in order to estimate the probability of the sample having come from each sub-population with a proportion of genetic markers from 0% to 100%. In this case the non-uniform distribution of true proportions in the population shown in Fig 1 would be very material to the investigation. As the sample size of selections from the population mystery box increased, it would converge eventually on the true proportion with the genetic marker in the population mystery box.

If the object of the exercise was to assess the probability of replication by predicting the content of the large sample mystery box, then the prior probabilities would be uniform and the initial posterior probabilities would be different to the posterior probabilities based on non-uniform population priors but again they would converge eventually on the true proportion of genetic markers in the mystery box. Therefore, the differences in the posterior probabilities of the true proportion in the mystery boxes would be greater for the initial small samples because it would be for these that the prior probabilities would have the greatest effect.

### 2.3. Probability notation

The scientist contemplating the large sample mystery box with an unknown proportion of ‘genetic marker present’ cards before starting the small random sample (e.g. of 99) would have an equal belief of about 1/101 = 0.0099 that it was from one of the 101 large sample boxes containing proportions from 0 to 100% because each large sample box contained the same number of cards (i.e. 100,000). As already indicated, one of these proportions from 0% to 100% is represented by *Θ*_*i*_ so that *Θ*_*i*_ can be any one of the proportions *Θ*_*i*_ from *Θ*_*1*_, *Θ*_*2*_, *Θ*_*3*_, up to *Θ*_*101*_. Therefore *i* can be any number in this case from 1 to 101 so that the prior probability of any one box having the unknown proportion *Θ*_*i*_, is *p(Θ*_*i*_*)* = 1/101 = 0.0099.

If the scientist selects 99 cards from the mystery box and finds that 50 have ‘genetic marker present’ written on them, then the probability of selecting this 50 out of 99 can be calculated by applying the binomial theorem (provided that the value of *Θ*_*i*_ is known or assumed). When the total number of cards selected is *n*, the number with genetic marker present on them is *r*, this observation is represented in this paper by [r\n]. Note that the reverse oblique \ denotes *r* observations out of *n* selections and not *r* divided by *n*).

The usual notation in probability theory is to regard capital *X* as the general outcome of observing the result of a random selection. The lower case *x* is a particular result of that selection’s general outcome *X*. This is expressed as *P(X = x)*. When x = 50, then the probability of selecting 50 randomly is expressed as *P(X = 50)*. Note that the number of selections n (e.g. 99) is merely implied on the left hand side of the equation and only appears explicitly on the right hand side in the calculation as: P(X=x)=p(x)=(nr)px(1−p)(n−x). This notation does not allow many of the details to be made explicit, so another notation will be used. Instead of *p* we shall use *p(Θ*_*i*_*)*. Instead of (nr) we use the underlying formula *n*!/*r*! * (*n*−1)!; instead of *p*^*x*^(1−*p*)^(*n*−*x*)^ we use *p*(*Θi*)^*r*^ * (1−*p*(*Θi*))^(*n*−*r*)^.

If the true proportion from which a selection is made is *Θ*_*i*_, then the likelihood of selecting the observed proportion [r\n] from *Θ*_*i*_ is:

$$p([r\backslash n]|\Theta i)=n!/r!*(n-r)!*{p(\Theta i)}^{r}*{\left(1-p(\Theta i)\right)}^{\left(n-r\right)}$$ Eq 1

The scientist can now calculate the likelihood of selecting [50\99] for each possible parameter value *Θ*_*i*_ from *Θ*_*1*_ to *Θ*_*m*_. Because each true parameter value *Θ*_*i*_ is equally probable before any samplings are made, the probability of each parameter *Θ*_*i*_ together with an observed selection result [r\n] will be:

$$p(\Theta i˄[r\backslash n])=p(\Theta i)*n!/r!*(n-r)!*{p(\Theta_{i})}^{r}*{\left(1-p(\Theta_{i})\right)}^{\left(n-r\right)}$$ Eq 2

This calculation is performed *m* times for each value of *Θ*_*i*_. If we sum the result *p*(*Θi* ˄ [*r*\*n*]) from Eq 2 for each *Θ*_*i*_ from *Θ*_*1*_ to *Θ*_*m*_ then this will tell us the proportion of times we would select [r\n] with ‘genetic marker present’ on the cards from all the boxes:

$$p(\left[r\backslash n\right])=\sum_{i=1}^{m}{p(\Theta }_{i}{)*n!/r!*(n-r)!}^{}*{p(\Theta_{i})}^{r}{*(1-p(\Theta_{i}))}_{}^{\left(n-r\right)}$$ Eq 3

Therefore, according to Bayes’ rule, the probability of seeing any particular *Θ*_*a*_ conditional on having selected [r\n] (i.e. *p*(*Θ*_*a*_|[*r*/*n*]) when *Θ*_*a*_ is a particular *Θ*_*i*_ to distinguish it from any other *Θ*_*i*_) can be found by dividing ${p(\Theta_{a})*\frac{n!}{r!}*(n-r)!}^{}*{p(\Theta_{a})}^{r}){*(1-p(\Theta_{a}))}_{}^{\left(n-r\right)}$ by $\sum_{i=1}^{m}p(\Theta_{i}){*\frac{n!}{r!}*(n-r)!}^{}*{p(\Theta_{i})}^{r}{*(1-p(\Theta_{i}))}_{}^{\left(n-r\right)}$ to give Eq 4:

$$p(\Theta_{a}|[r\backslash n])=\frac{{{p(\Theta }_{a})*\frac{n!}{r!}*(n-r)!}^{}*{p(\Theta_{a})}^{r}{*(1-p(\Theta_{a}))}_{}^{\left(n-r\right)}}{\sum_{i=1}^{m}p(\Theta_{i}){*\frac{n!}{r!}*(n-r)!}^{}*{p(\Theta_{i})}^{r}{*(1-p(\Theta_{i}))}_{}^{\left(n-r\right)}}$$ Eq 4

### 2.4. Normalisation

The process of applying Eq 4 not only to one value of *Θ*_*a*_ but to each value *Θ*_*i*_ in a distribution from 1 to 101 is usually described as its normalisation. This will provide the posterior probability of each possible true result from *Θ*_*1*_ *to Q*_*101*_. This means that they will sum to one (i.e. $\sum_{i=1}^{m}p(\Theta_{i}|[r|n])=1$). Normalisation is also applied to likelihood density distributions, including those of Gaussian distributions where the likelihood values based on the binomial distribution are not probabilities (as in the above cases based on the binomial distribution).

### 2.5. The probability of replication within a specified range

The probability of precisely replicating a result may be so small as to be not very helpful. In order to have a meaningful result, it is usually necessary to calculate the probability of the true result falling within a range. The range chosen will usually be of special significance to a scientist in that it may be used to accept or reject some scientific hypothesis. It may be a range with a single bound (e.g. the probability that the long term replication range will be greater than a proportion of 50%). The range may also be a difference between proportions or means greater than zero. It may be a range with two bounds (e.g. between 40% and 60%). It may also be range that is based on the observed result (e.g. a range which contains 47.5% of true results above and below the expected outcome that is equal to the observed result so that it contains 2*47.5 = 95% of all results). (Note that * is used as a multiplication sign as in a spreadsheet). If we wish to find the probability of such a range of true results (e.g. from *Θ*_41_ = 40% *to Θ*_61_ = 60%), then we can add the probabilities of each parameter within that range (e.g. $\sum_{i=41}^{61}p(\Theta_{i}|[r\backslash n])$)

### 2.6. Combining two study results

If we took samples in two stages (e.g. [9\12] and [21\37] and combined them to give [(9+21)\(12+37)] = [30\49]) then we can estimate the probability of a particular parameter *Θ*_*a*_ conditional on this pair of results in different ways. In order to put matters in general terms we will call the pair of samples [r_1_\n_1_] (e.g. [9\12]) and [r_2_\n_2_] (e.g. [21\37]).

If we calculate:

$$\left[p([r_{1}\backslash n_{1}]|\Theta_{i}))=n_{1}!/r_{1}!*(n_{1}-r_{1})!*{p(\Theta_{i})}^{r_{1}}*{\left(1-p(\Theta_{i})\right)}^{{(n}_{1}-r_{1})}\right]$$ Eq 5

$$and\;\left[p\left(\left[r_{2}\backslash n_{2}\right]|\Theta_{i}\right)\right)=n_{2}!/r_{2}!*\left(n_{2}-r_{2}\right)!*p{\left(\Theta_{i}\right)}^{r_{2}}*{\left(1-p\left(\Theta_{i}\right)\right)}^{\left(n_{2}-r_{2}\right)}]$$ Eq 6

then if the likelihoods of both samples are statistically independent:

$$p([r_{1}\backslash n_{1}]˄[r_{2}\backslash n_{2}]|\Theta_{i})=p([r_{1}\backslash n_{1}]|\Theta_{i})*p([r_{2}\backslash n_{2}]|\Theta_{i})$$ Eq 7

If for each i = 1 to m, we perform the sum $\sum_{i=1}^{m}\;\{p(\Theta_{i})*p(\left[r_{1}\backslash n_{1}\right]|\Theta_{i})*p(\left[r_{2}\backslash n_{2}\right]|\Theta_{i})\}$ then this will give us the prior probability *p*([*r*_1_\*n*_1_]˄[*r*_2_\*n*_2_]). If we divide the product for a single true result of *Θ*_*a*_ e.g. {*p*(*Θ*_*a*_) * *p*([*r*_1_\*n*_1_]|*Θ*_*a*_) * *p*([*r*_2_\*n*_2_]|*Θ*_*a*_)} by the prior probability *p*([*r*_1_\*n*_1_]˄[*r*_2_\*n*_2_]) we will get *p*(*Θ*_*a*_/[*r*_1_\*n*_1_]˄[*r*_2_\*n*_2_]) which will be the same as *p*(*Θ*_*a*_/[*r*\*n*]). For example, if *Θ*_*a*_ is 47%, then *p(47%****|****[30\49]) = p(47%****|****[9\12]˄[21\37])*. In this situation, *p*(*Θ*_*a*_/[*r*_1_\*n*_1_]) can be regarded as either a Bayesian or Frequentist non-marginal prior probability, *p*([*r*_2_\*n*_2_]|*Θ*_*a*_) as a likelihood probability and *p*(*Θ*_*a*_/[*r*_1_\*n*_1_]˄[*r*_2_\*n*_2_]) as the posterior probability.

### 2.7. Comparisons with Gaussian distributions

In order to compare the results of estimating the probability of replication by using both the binomial and Gaussian distributions, the likelihood of observing any [r_i_\n] was estimated by first calculating the standard error *σ* = (*p*(*Θ*_*nh*_) * {1−*p*(*Θ*_*nh*_)}/n)^1/2^ (when *Θ*_*nh*_ is the null hypothesis in this case for example). The probability of a particular [r_k_\n] based on the Gaussian likelihood (omitting the constant *1/√2π* in the numerator and denominator as they cancel out) is:

$$p(\left[r_{k}\backslash n\right]|\Theta_{nh})=\frac{exp(-0.5*{\left(\frac{\frac{r_{k}}{n}-\Theta_{nh}}{\sigma }\right)}^{2})}{\sum_{i=1}^{n}exp(-0.5*{\left(\frac{\frac{r_{i}}{n}-\Theta_{nh}}{\sigma }\right)}^{2})}$$ Eq 8

### 2.8. The probability of impeccable reproducibility

The concept of long term replication means that that the sampling methods used for the initial observations will have to be continued in exactly the same way until the very large sample is complete. If the process is conducted by different individuals or teams then the methods will have to be described impeccably and repeated impeccably. It is also important that the way in which subjects of the study is selected does not change inadvertently. If there is some known change, then the calculations based on an assumption of long term random sampling model will be invalid. They will also be invalid if some methodological irregularities are actually discovered (e.g. cherry picking of results or some other form of cheating).

In many circumstances, methodological irregularities or external events that are beyond the control or knowledge of the scientist may not be obvious but suspected. Such hidden changes might be more common in some disciplines than others. For example some confounding event may be suspected with a probability of 0.9 according to an informal estimate. This might translate into an expectation that in a group of such identical studies, impeccable reproducibility may only happen in say 90% of studies.

If we consider a series of studies with the result of [50\100] of which 90% are impeccably reproducible, then only 0.9*100 = 90 on average can be relied upon to have been selected in a random way out of 100 to give a guaranteed result of 0.9*50 out of 0.9*100 to give [45/90]. The remaining unreliable 10 could have any biased result ranging from [0/10] to [10/10], so that for the 100 random samples, the overall result in the long term could be between [(45+0)\(90+10)] = [45\100] and [(45+10)\(90+10)] = [55\100]. In order to express a sense of uncertainty about the impeccability of the selection process on long term replication, we could display three curves showing the range of the probabilities of realistic replication based on the above possible sampling results of [45\100], [50\100] and [55\100].

If *p(IR|MA)* represents the probability of Impeccable Reproducibility (IR) conditional on Methodology Assessment (MA) and the observed result is [r\n], then the range of outcomes of a binomial likelihood distribution will lie between [{*p*(*IR*|*MA*) * *r*}\*n*] (e.g. [0.9*50\100] = [45\100]) and [{2−*p*(*IR*|*MA*) * *r*}\*n*] (e.g. [(2–0.9)*50\100] = [55\100]). For the special case of [r\n] = [50\100], the calculation of the curves can be simplified by shifting the cumulative distribution curve for [r\n] = [50\100] to the right and left by *r*(+/−*r* * {1−*p*(*IR*|*MA*)}) (e.g. 50–50*(1–0.9) = 45 or 50 + 50*(1–0.9) = 55). Moreover, when *r/n* is 0.5 the distribution will be symmetrical and can be modelled accurately with a Gaussian distribution so that two upper and lower range curves for a Gaussian distribution can also be plotted in the same way. This could be applied to continuous variables if the data are expressed in terms of Z scores. A detailed example will be given in section 3.9. It must be emphasised that this is merely one proposal for estimating the realistic probability of replication based on an informal estimate of a probability of impeccable reproducibility and a rigorous idealistic probability of replication.

## 3. Results

### 3.1. The prior probability of true proportions

The choice of true intervals between 0% and 100% depends upon the calculation accuracy required. In the initial example the interval was 1%, so that there were 101 points (0%, 1%, 2%, … 99%, 100%). The probabilities of selecting [50\99] were calculated for each of these hypothetical true proportions (e.g. if the true value *Θ*_*i*_ is 60%, the likelihood probability of selecting [60\99] = 99!/60! * (99−60)! * 0.6^60^ * (1−0.6)^(99−60)^ = 0.0812 [13].

This calculation was performed for all the hypothetical true values from 0% to 100%. When each of these 101 likelihood probabilities was multiplied by the uniform prior probability for the 101 true values their sum was 1, the uniform prior probability therefore being 1/101 = 0.0099. However, for an observed proportion of [30\49] the sum was 2, suggesting a uniform prior probability for *n* = 49 of 2/101 = 0.198 0. For an observed proportion of [21/37] the sum was 2.6316 suggesting a prior probability for *n* = 37 of 2.6316/101 = 0.02606.

If these priors are correct, then the likelihood probability of observing [50\99] or [30/49] or [21/37] conditional on the true parameter of 50% should be equal to the posterior probability of the true parameter conditional on selecting [50\99] or [30/49] or [21/37] respectively based on the binomial distribution. However, this was not so. They are only equal if the prior probability of each true parameter *p(Θ*_*i*_*)* is also set at 0.01 instead of 0.0099. In other words for *n* selections, the prior probability is 1/(*n*+1) and when *m* true proportions are being considered, their uniform prior probability is 1/(*m*-1). When *n* = 49 the uniform prior probability of p([r\49]) is 2/100 = 0.02 and when *n* = 37 then the prior probability of p([r\37]) is 2.63/100 = 0.0263. This suggests that it is the 100 spaces or bounding pairs of true values that determines the value of the prior probability *p(Θ*_*i*_*)*.

### 3.2. The effect of choosing different intervals for true proportions

Instead of the 101 possible true proportions forming the baseline, if we had specified 1001 data points (i.e. 0.0%, 0.1%, 0.2% up to 99.9%, 100.0%), then the prior probability of each possible true proportion would have been 1/1000 = 0.001 instead of 0.01. The likelihood probability of selecting [40\99] from *Θ*_*401*_ = 0.40% (when *p(Θ*_*401*_*)* = 0.001) would have been *0*.*0812* [13] and similar to the likelihood probability of choosing [60\99] from *Θ*_*61*_ = 60% (when *p(Θ*_*61*_*)* = 0.01). The posterior probability of seeing *Θ*_*61*_ = 60%) (with a prior probability of 0.01) would be the same at *0*.*0812* [13]. However, the posterior probability of seeing *Θ*_*401*_ = 40.0% (with a prior probability of 0.001) conditional on an observation of [40\99] would be 1/10^th^ of the likelihood: i.e. *0*.*00812* [13]. This is because the prior probability of selecting [40\99] is 0.01 and the prior probability of a true result of *Θ*_*401*_ = 40.0% is 0.001, so the posterior probability of *Θ*_*401*_ = 40.0% is 0.001*0.*0812*/0.01 = *0*.*00812*.

**Fig 2:**
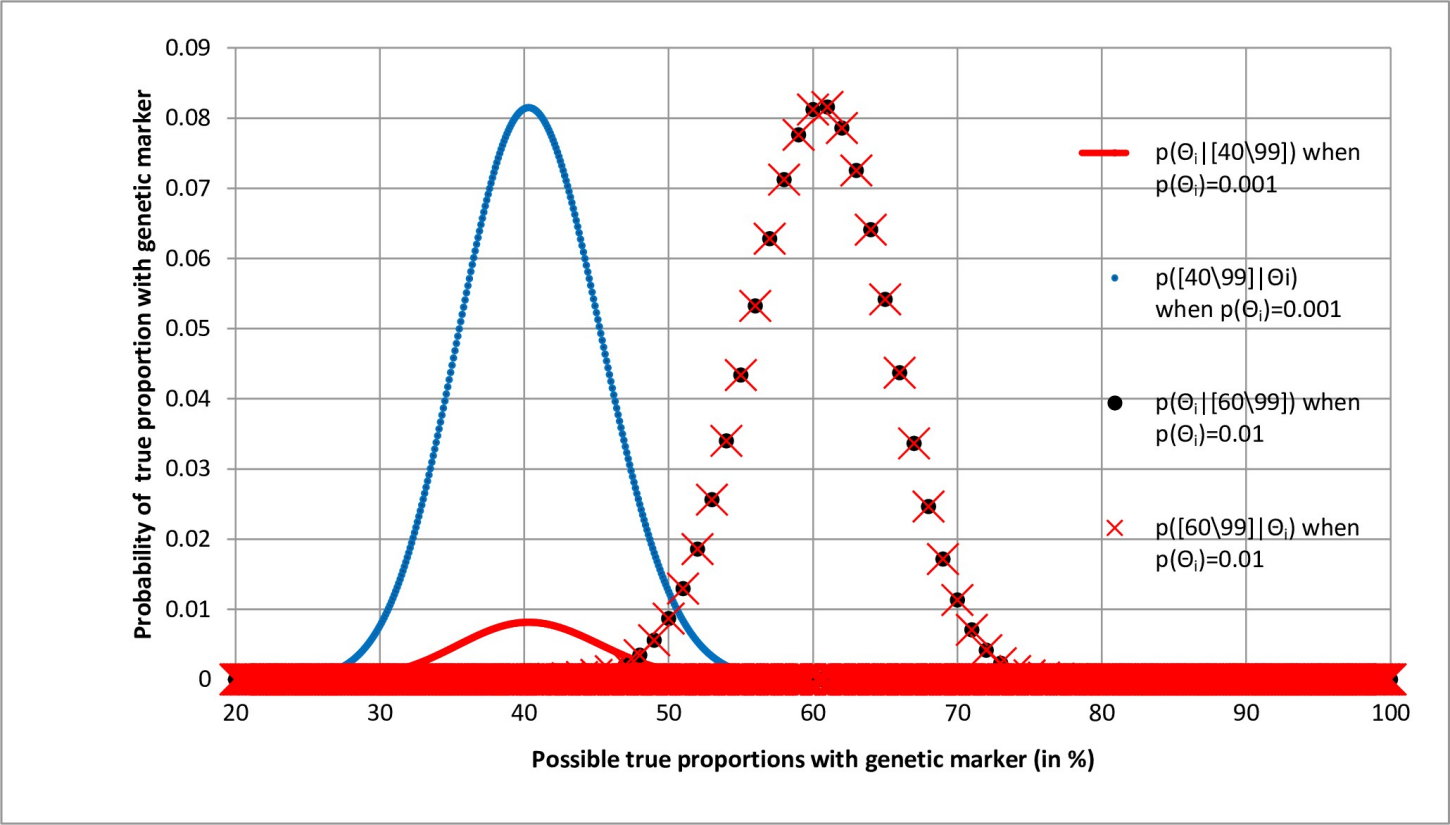
Comparing effects of prior probabilities of 0.01 and 0.001 for true proportions on the likelihood and posterior distributions of observed proportions of [40\99] and [60\99].

The overall results of using intervals of 0.01 and 0.001 are shown by the four curves in Fig 2. The curve markers represent actual likelihood and posterior probabilities. The curves with modes at 40% are based on 1001 points with intervals of 0.001.The taller curve represents the likelihood distribution, the maximum probability at the mode being *0*.*0815* [13]. The shorter curve represents the posterior distribution, the maximum posterior probability at the mode being 1/10^th^ of the likelihood at *0*.*00815* [13]. The two curves with modes at 60% are based on 101 points with intervals of 0.01 and are superimposed. One curve represents the likelihood distribution, the maximum probability at the mode being *0*.*0816* [13]; the other curve represents the posterior distribution the maximum posterior probability at the mode being the same at *0*.*0816* [13].

Although the posterior probabilities of true proportions will be different when their priors are different, the posterior probabilities of ranges of true proportions should be similar, especially if they are broad (e.g. from 55% to 65% as opposed to 49% to 51%). Differences will be due to rounding errors or because of differences in the precision of the estimates and will be greater if data points are sparse within the specified range. When 1001 points are used to construct the curves on the left of Fig 2, the sum of posterior probabilities from 30% to 50% is *0*.*960* [13]. When the range is widened slightly so that it is from 29.5% to 50.5%, the sum is higher at 0.969 [13]. For the curve based on 101 points, the sum of posterior probabilities from 50% to 70% is 0.971 [13] but when the range was widened slightly from 49.5% to 70.5% the sum remained the same at 0.971 [13]. This is because unlike the curve based on 1001 points, the curve based on 101 points had no points between 49% and 50% or between 70% and 71% that would change the sum. Clearly it is best to set up calculations and curves with as many true points as necessary in order to achieve a desired accuracy when estimating the probabilities of true ranges.

### 3.3. Probability pairs

The expected true probability corresponding to an observed sample of [50\99] is 0.505, which is not represented by any of the true values from 0%, 1%, 2% up to 99% and 100%. Instead, the expected true value is represented by the pair 0.50 and 0.51. It is interesting to note therefore that [50\99] appears to map to what has been described as an ‘induced probability pair’ [14]. This pair allows the original observation to be calculated, in this case the observed proportion of [50\99]. Thus when the upper proportion is *P*_*u*_ (e.g. 0.51) and the lower proportion is *P*_*l*_ (e.g. 0.50), the originating denominator can be recalculated as *1/(P*_*u*_*-P*_*l*_*)-1* = 1/(0.51–0.50)-1 = 1/0.01–1 = 100–1 = 99. The numerator is *1/(P*_*u*_*-P*_*l*_)*P1 = (1/(0.51–0.50))*0.50 = 50. The other interesting property of the pair is that they form a chain (e.g. {0.50, 0.51}; {0.51, 0.52}; {0.53, 0.53}; etc.) so that there are no gaps although they are composed of discrete values. However, as the number of chosen true values become very large (e.g. 1,000,000) the paired values become very close so that [50\99] maps to the pair 0.505050, 0.505051. The way in which the precision of probability estimates can be controlled by the number of data points is analogous to the way increasing the number points on a ruler improves the accuracy for measuring lengths.

When the uniform prior probability of each true value from 0% to 100% is set at 0.01 then the posterior probabilities of the true values are equal to their likelihood probabilities based on a sample of 99, which is a useful property for exploring these concepts such as the relationship between binomial distributions and posterior probabilities based on normalised likelihood distributions.

### 3.4. Comparing binomial, normalised binomial likelihood and Gaussian distributions

**Fig 3:**
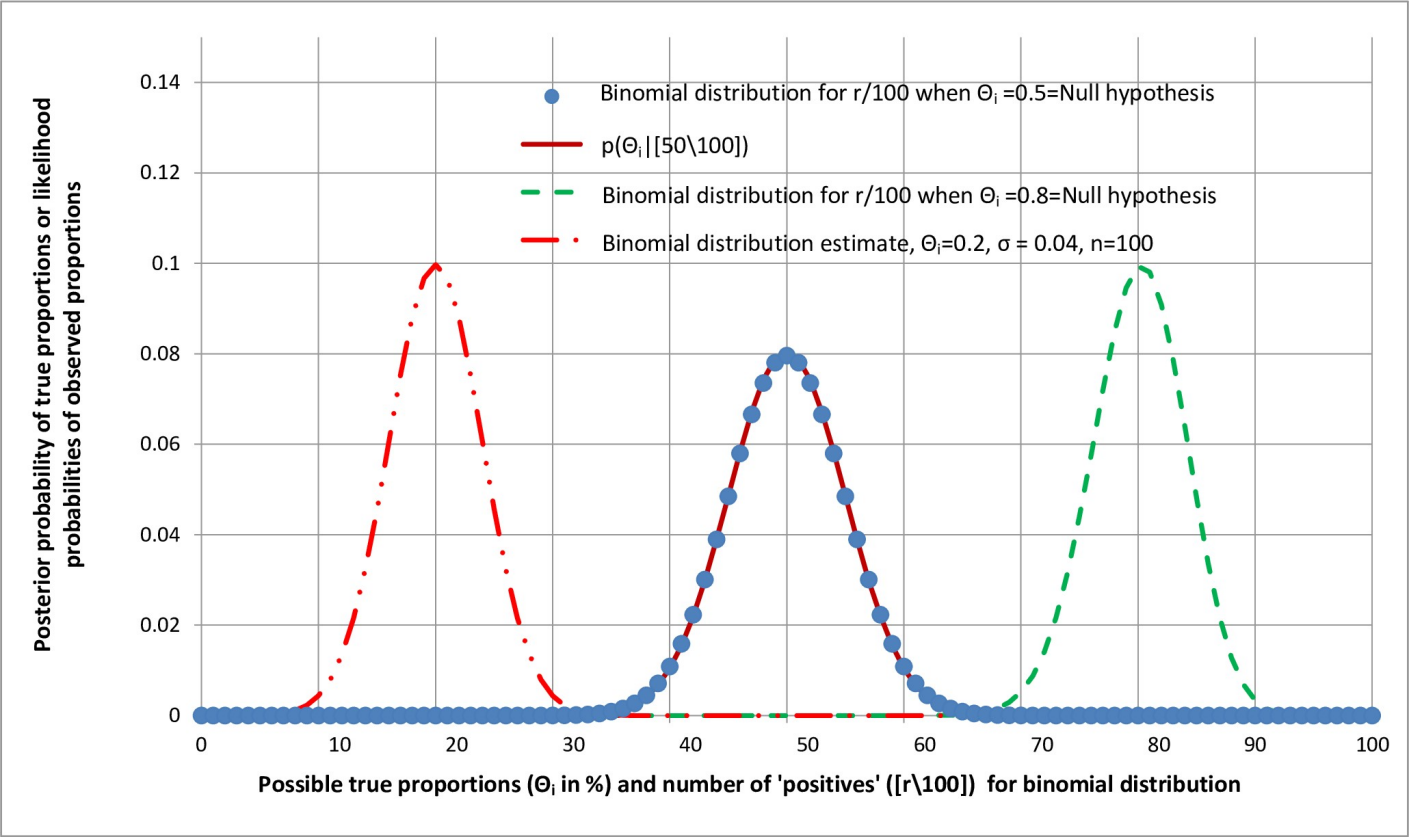
Comparison of distributions of posterior probabilities of true proportions conditional on observed proportions and binomial and Gaussian likelihood distributions.

The posterior probability distribution for an observation of 50/100 might be expected to be similar to the binomial distribution created with 100 possible outcomes selected at random from a population with a proportion of 0.5. This binomial probability distribution is represented by the round solid markers in Fig 3. The binomial and the normalised binomial likelihood distributions are indeed superimposed, at least when the mode of the distributions is near to 0.5 and they are both symmetrical. This also suggests an alternative interpretation for replication.

The correspondence between the binomial and the normalised posterior distributions could be explained by 0.5 being the pooled proportion of a very large number of samples of [50\100] from different studies. If all these very large number of studies were repeated with 100 observations, then the distribution of results from [0\100] to [100\100] could be regarded as random selections from large pooled population with a proportion of 0.5 and modelled by the binomial distribution displayed in Fig 3. This would provide an alternative interpretation for replication. It would be the proportion of times a result would fall within a range that includes the original result when a study is repeated with impeccable reproducibility and with the same number of observations (e.g. 100). However, the result would be less accurate than that provided by the posterior probability based on normalising a binomial likelihood distribution as any large number of true values (e.g. 100,000) can be used for the latter to give very accurate estimates, whereas from the binomial distribution the accuracy would be limited to the number of observations in the study (e.g. 100). The results of the two methods would only coincide if the number of true values were the same as the number of observations when applying the binomial theorem (e.g. as in Fig 3).

### 3.5. The null hypothesis

The proportion 0.5 can be regarded as a null hypothesis from which can be calculated the likelihood probability of each possible observation from [0\100] to [100\100]. If the null hypothesis had been 0.5 then the probability of observing [40\100] or something more extreme (i.e. [39\100], [38\100], etc.) would be the sum of the values in the tail at [40\100] and beyond. It would be a one-sided P-value of 0.0284 in this case [13]. This would be similar to the probability of replication at the null hypothesis or something more extreme when a sampling observation of [50\100] had been made. It was 0.0265 [13].

It is noticeable from Fig 3 that when the observed proportion is 50% (e.g. [50\100]) then the posterior probability distribution based on a normalised binomial likelihood is symmetrical. This is also the case for the superimposed binomial distribution, which means that the one-sided P-value is very similar to the probability of non-replication values up to 40% (i.e. equal to or less than 40%). The term non-replication is used here because replication implies that the probability of a range of true values that includes the observed value is high. Also non-replication includes the null hypothesis or a true value more extreme in order to make comparisons with the one-sided P-value.

When the observed true values are very different from 50% (e.g. *Θ*_*i*_ = 80% as in the right hand binomial distribution in Fig 3) the distributions become skewed. The proportion in the tail of the binomial distribution at [90\100] or something more extreme is 0.0057 [13] of the total distribution when *Θ*_*i*_ = 80% but the tail at 70% or something more extreme is different at 0.0112 [13] of the total distribution. This illustrates that when *Θ*_*i*_ ≠ 50%, the binomial distribution is asymmetrical. However for the Gaussian model of the binomial distribution when *Θ*_*i*_ = 20% on the left hand side of Fig 3, the tail at [10\100] or below is 0.0086 [13] of the total distribution; the tail for [30\100] or above also being exactly 0.0086 [13] illustrating that the Gaussian distribution is symmetrical for all values of when *Θ*_*i*_ from 0% to 100%.

The symmetry or otherwise of distributions is important because when the binomial or normalised binomial likelihood distributions are not symmetrical, there may be loss of correspondence between the P-value and the probability of non-replication. It should also be noted that when the symmetrical Gaussian distribution is used to model the binomial distribution, then it provides an accurate representation when the latter is also symmetrical (i.e. when *Θ*_*i*_ = 50%). However, the Gaussian likelihood distribution will also be symmetrical at proportions near to 1 or 0 (e.g. when *Θ*_*i*_ = 20% on the left of Fig 3).

### 3.6. The relationship between the probability of replication and P-values

**Fig 4:**
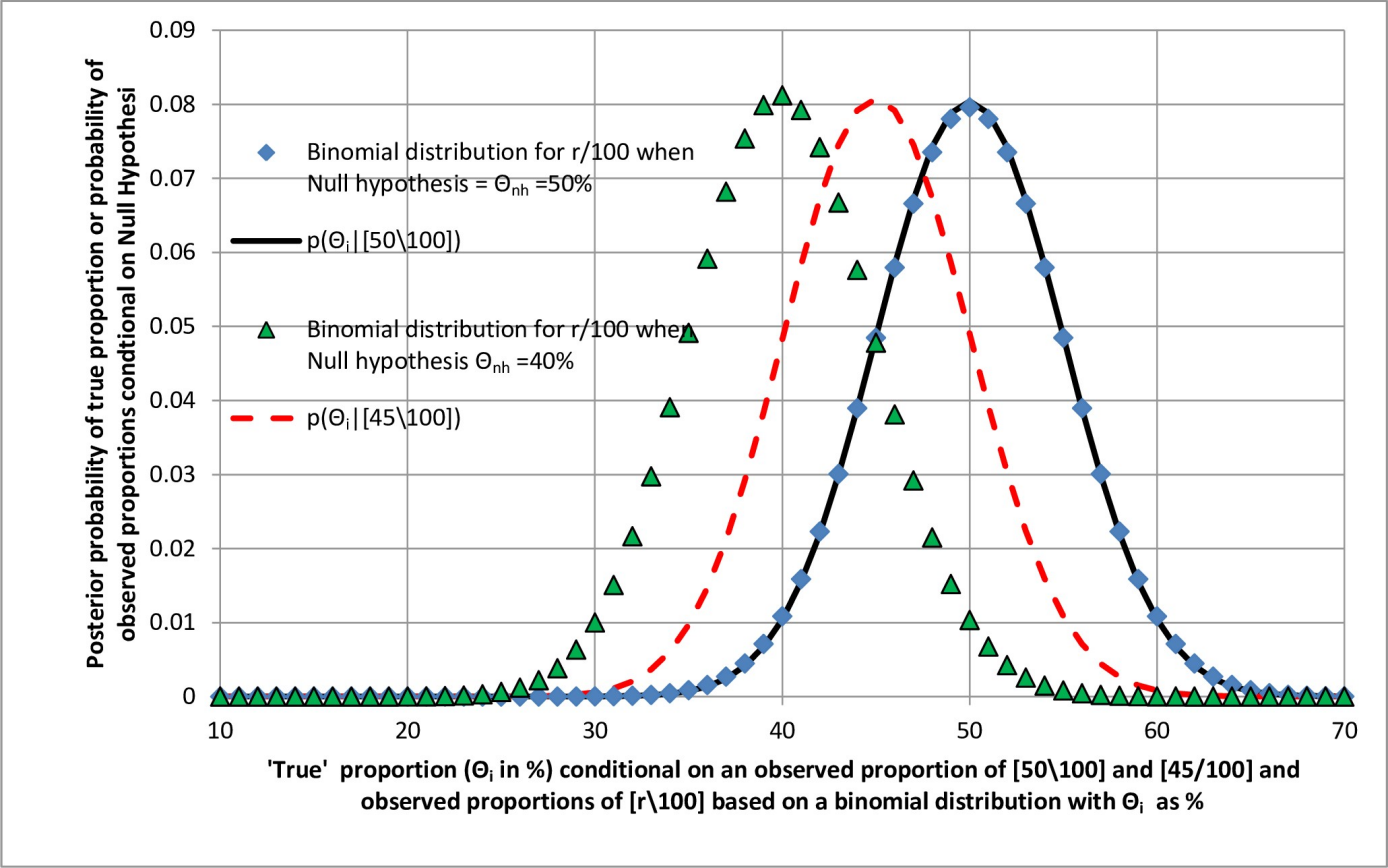
Comparing the probability of non-replication conditional on an observed proportion and one-sided P-values conditional on Null Hypotheses.

Fig 4 displays two binomial distributions and two posterior probability distributions based on normalised binomial likelihood distributions. The binomial distribution for the null hypothesis of 50% (the round markers) and the posterior probability distribution conditional on an observation of [50\100] (the continuous line) are both symmetrical and superimposed.

The binomial distribution for the null hypothesis of 40% (the triangular markers) and the posterior probability distribution conditional on an observation of [45\100] (the broken line) are both non-symmetrical, the tails for the lower values being shorter than for the higher values. The probability of non-replication of a true proportion of 50% or above (corresponding to the upper tail of the red broken curve distribution beyond 50%) conditional on an observation of [45\100] is 0.1852 [13]. For a null hypothesis of 50%, the one-sided P-value for [45\100] or something more extreme (corresponding to the lower tail of the round marker distribution beyond 50%) is very similar at 0.1841 [13]. The difference between them is 0.0011. However, the probability of non-replication with a true proportion of 40% or below conditional on an observation of [45\100] is 0.1752 [13]. For a null hypothesis of 40% the one-sided P-value for [45\100] or something more extreme (i.e. higher) is 0.1789 [13]. The difference between them is 0.0037. Note that the latter difference of 0.0037 is greater than the difference of 0.0011 when the null hypothesis was 50% and the associated binomial distribution was symmetrical.

**Fig 5:**
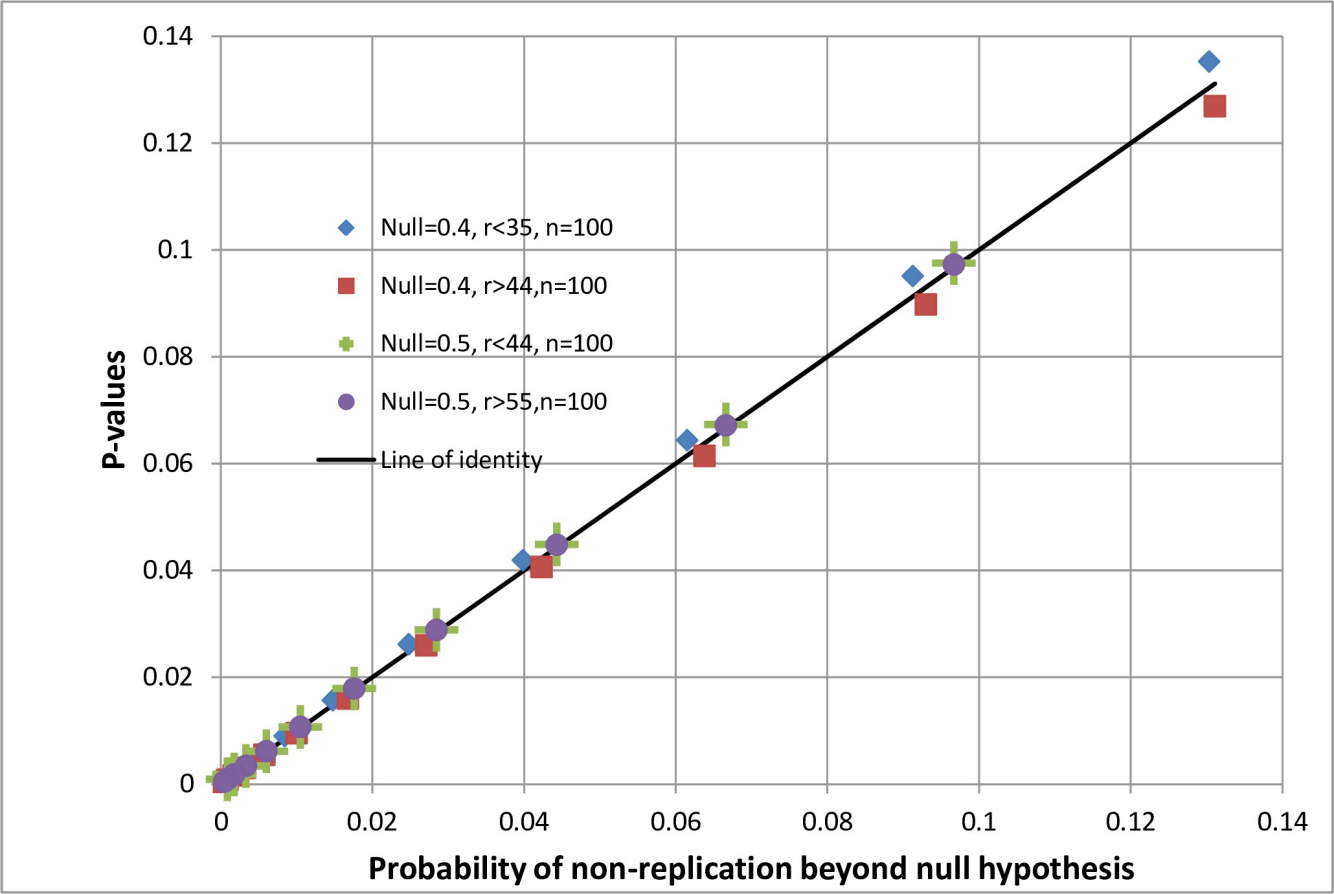
Comparison of probabilities of non-replication for observed proportions of r/100 with one-sided P-values for different null hypotheses of 0.5 and 0.4.

Fig 5 displays the relationship between one-sided P-values and probabilities of non-replication for the null hypotheses of 50% and 40% for a range of observations from [34\100] to [66\100]. Of the latter, Fig 4 shows the posterior distributions only for [45\100] and [50\100] and the binomial distributions only for *Θ*_*i*_ = 50% and *Θ*_*i*_ = 40%). For the range of distributions, it can be seen from Fig 5 that for the null hypothesis of 50% (with a symmetrical binomial distribution) there is a much closer relationship between the probability of non-replication and the P-value than when the null hypothesis is 40% with a non-symmetrical binomial distribution. This demonstrates that when the binomial distribution based on the null hypothesis is symmetrical then the probability of non-replication is closer to the one-sided P-value. If the null hypothesis’s binomial distribution is not symmetrical but slightly skewed there is a greater difference between the one-sided P-value and the probability of non-replication. In these examples the posterior distribution of true values conditional on an observation of [45\100] (the dotted line) is skewed which would be why the probability of non-replication at 40% and lower is not identical to the one-sided P-value for an observation of [45\100] or higher based on a null hypothesis of 40%.

With the exception of distributions centred on 50%, the others in Fig 4 are not symmetrical because they are based on the binomial theorem and constrained by the limits of 0% and 100%. This is the result of using proportions as examples (but at least they can be calculated exactly using the binomial distribution without potentially misleading assumptions). However, when Gaussian distributions are used to model continuous variables without the constraints of upper and lower limits, then the distributions of observations based on null hypotheses and the posterior probability distributions of true values should be symmetrical so that the one-sided P-values and probabilities of true values based on some observation should be the same. This depends on the assumption that the data can be modelled appropriately by a Gaussian or some other symmetrical distribution of course.

### 3.7. The relationship between two sided P values and the probability of replication

If 60% was the null hypothesis, then the P value for [50/100] or something below ([49\100] etc.) would be 0.0271 [13]. Similarly If 40% was the null hypothesis, then the P value for [50/100] or something above ([51\100] etc.) is 0.0271 [13]. The two sided P-value would be 0.0271*2 = 0.0542. For an observation of [50\100] the probability of non-replication at 40% or below and 60% and above would be 0.0265 [13] so the probability of non-replication at 40% and below and 60% and above would be 0.0265*2 = 0.0530. This would correspond to the two-sided P-value of 0.0542. However the probability of replication between 40% and 60% exclusive would be 1–0.0542 = 0.9458. A 95% confidence interval would include 40% and 60%, its limits would be 39.84 and 60.16 [13]. This confidence interval would correspond to a probability of replication within the range of 39.84 and 60.16 of 0.95. These two-sided replication ranges would be used when the object was to show that the range was narrow. There is therefore a very close relationship between confidence intervals, one and two sided P values and probabilities of replication provided that the distributions used to calculate them are symmetrical.

### 3.8. Combining probability distributions using Bayes’ rule

**Fig 6:**
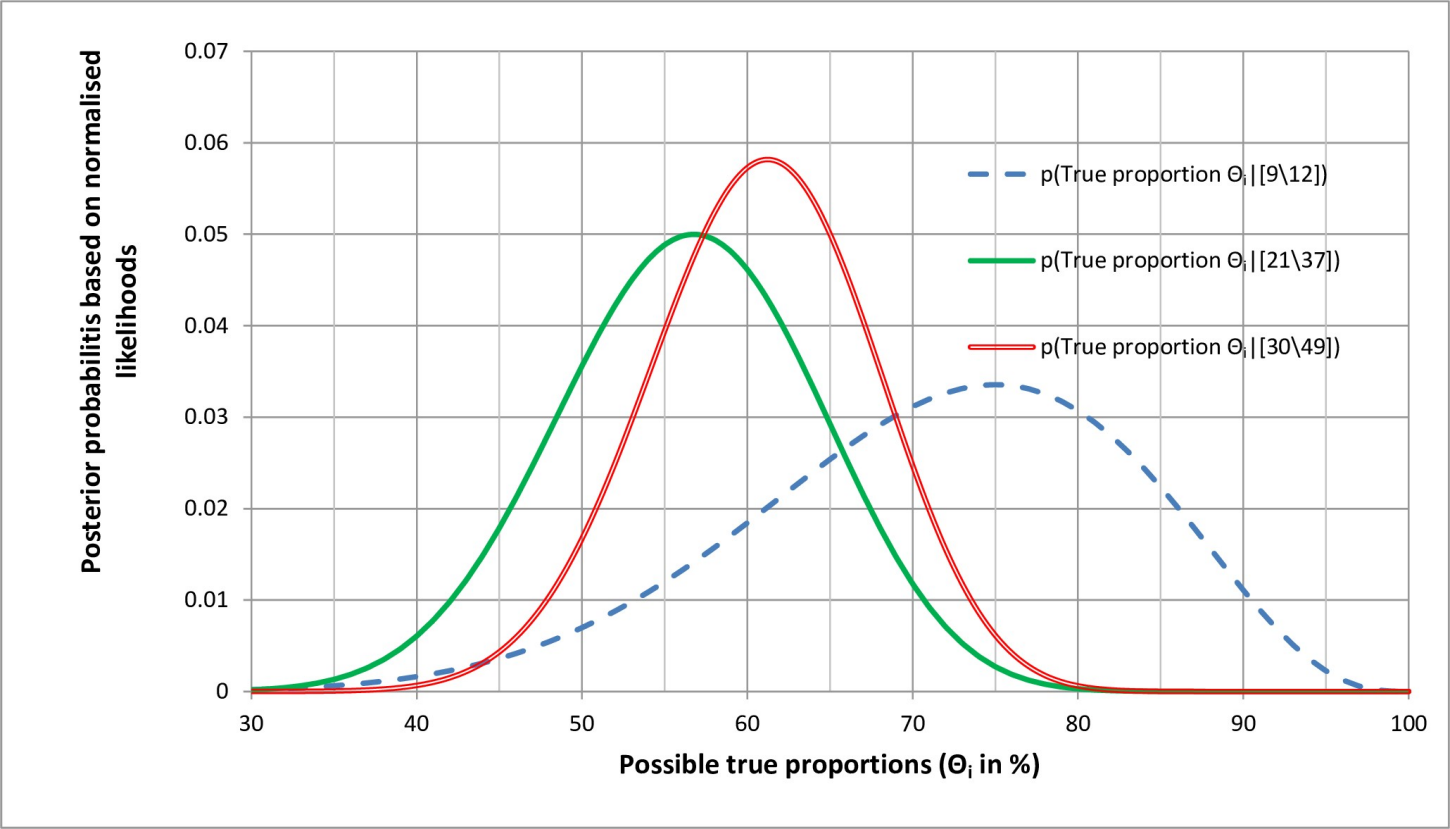
Calculating the posterior probability distribution of true outcomes from a prior probability distribution and a likelihood distribution *Θ*_*i*_.

Fig 6 shows three distributions. The dotted line represents a prior probability distribution obtained by normalising the likelihood distribution for [9\12]. It is prior in the sense that it prior to combining with another distribution and not a marginal prior distribution. The marginal prior distribution is uniform with prior probabilities of 0.01. The solid line represents the normalised likelihood distribution for [21\37] and the double line a posterior distribution conditional on [30\49] obtained from the product of the prior probabilities for the broken line [9\12] distribution and those of the solid line [21\37] distribution and then normalising the results of this product [13]. The double line posterior distribution can also be obtained by normalising the likelihood distribution for [(9+21)\(12+37)] = [30\49] [13], the latter representing a simple meta-analysis. The result of latter, combining the distributions for [9\12] and [21\37] is the same when either or both are likelihood distributions, or posterior distributions created by normalising the likelihood distributions. The reason for this is that when the marginal or base-rate prior probabilities are uniform, their likelihood ratios and odds are the same. The products of the likelihood distributions for [9\12] and [21\37] will not be the same as the likelihood distribution for [30\49]. However, when that latter distribution is normalised it will provide the same posterior probability distribution as that obtained by normalising the product of the two likelihood distributions for [9\12] and [21\37].

### 3.9 Marginal Bayesian priors and conditional prior probabilities

It has been emphasised already that the marginal prior probability of some phenomenon in a sub-population is immaterial to the result of random sampling directly from that sub-population for estimating the probability of replication. When a scientist wishes to estimate the probability of replication, it is the result of an ideally large set of study observations that she or he needs to predict. If we term the marginal uniform prior probabilities of 0.01 in Fig 6 as primary prior probabilities then these are combined with the likelihood distribution for [9\12] to create a posterior distribution. This posterior distribution based on [9\12] then becomes a secondary non-marginal prior probability distribution to be combined with another likelihood distribution of [21\37] to create a 2^nd^ posterior probability distribution conditional on the combined observation of [9\12]^[21\37] = [30\49]. However, it does not matter if someone thought that a prior distribution such as that representing the likelihood distribution for [9\12] in Fig 6 was a marginal primary or non-marginal secondary prior probability distribution; the result of the calculation would be the same.

The result of combining a Bayesian marginal prior probability distribution based on an imagined observation of [9\12] with the likelihood of a real observation of [21\37] would therefore be the same as that described in the preceding paragraph. This is because care is always taken to ensure that a Bayesian marginal prior probability is arrived at without knowing the subsequent observation used to create a posterior probability. This means that the implied evidence on which the Bayesian prior probability is based could be regarded as being statistically independent conditional on the possible true results. Because of this, the result of the Bayesian calculation would be the same as if it was assumed that it was based on a primary unconditional prior probability or a secondary conditional prior probability with underlying uniform unconditional primary marginal prior probability.

The point was made in section 2.2 that the prior probability distribution has less influence on a posterior probability distribution as more data is obtained to increase a sample size. The population genetic marker frequencies in Fig 1 were based on the likelihood distribution of [r\n] = [1\3] so that this would be a conjugate distribution with respect to another observed proportion such as that of [50\99]. If this prior distribution based on [1\3] were combined with [50\99] to give [51\102] the resulting posterior distribution would be little different to the posterior distribution generated by [50\99] alone.

### 3.10. The reproducibility of study methods

**Fig 7:**
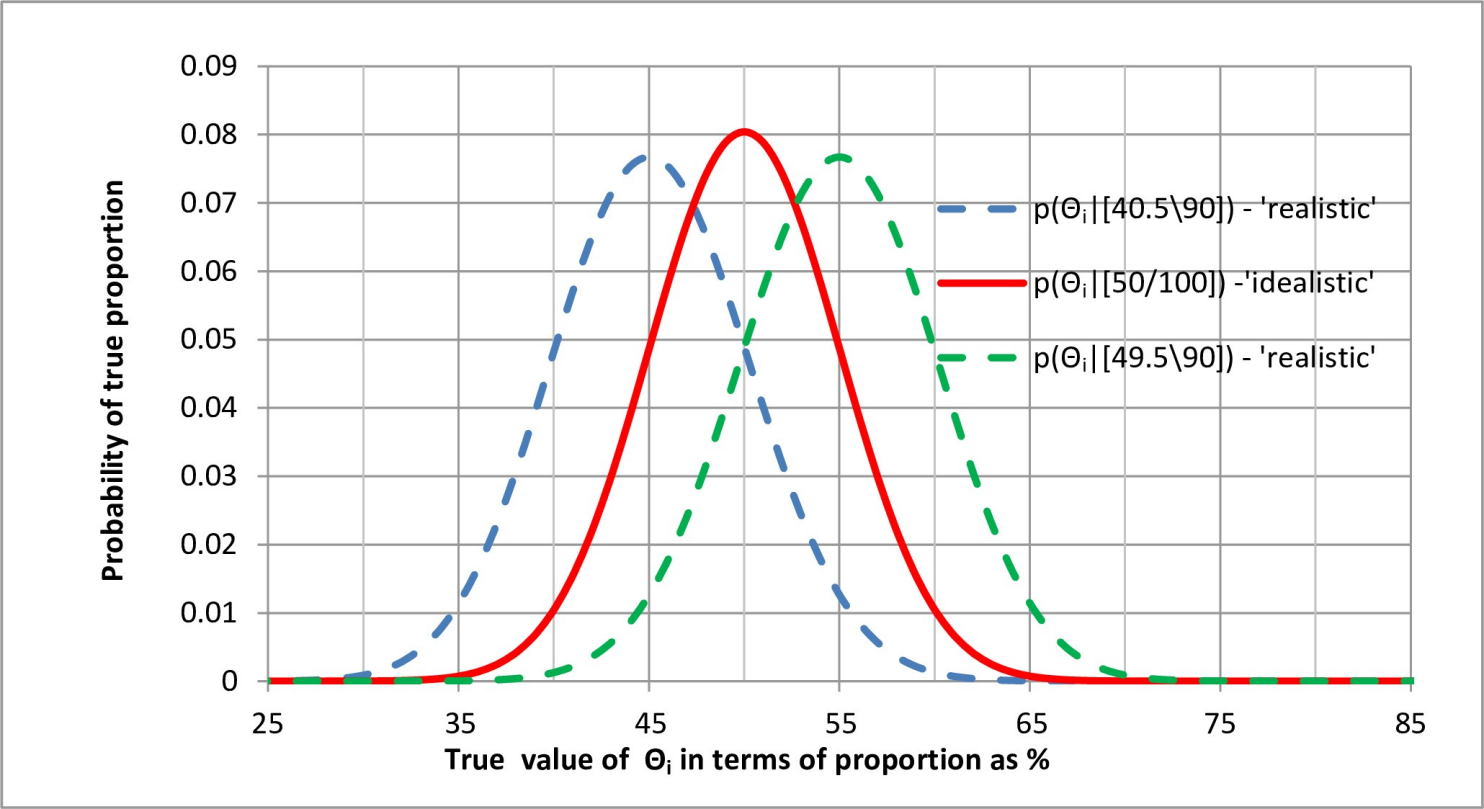
Taking into account the probability of impeccable reproducibility for an observation [50\100].

If an observation of 50 people with some feature (e.g. the presence of a genetic marker) were made out of a total of 100 in a large number of different studies, but there was only a probability of 0.9 that any one of these studies had been described and continued impeccably, then only 0.9 of the studies can be assumed to have been continued impeccably giving a reliable result of [0.9*50\0.9*100] = [45\90]. The remaining 10 observations could have any biased result from [10\10] to [0\10]. The result of the observations in the long term could range from [45\100] to [55\100] with an expected result of [50\100] if we assume that the imperfections had no effect on the result after all. We could therefore plot three posterior probability distributions based on the above results as shown in Fig 7.

**Fig 8:**
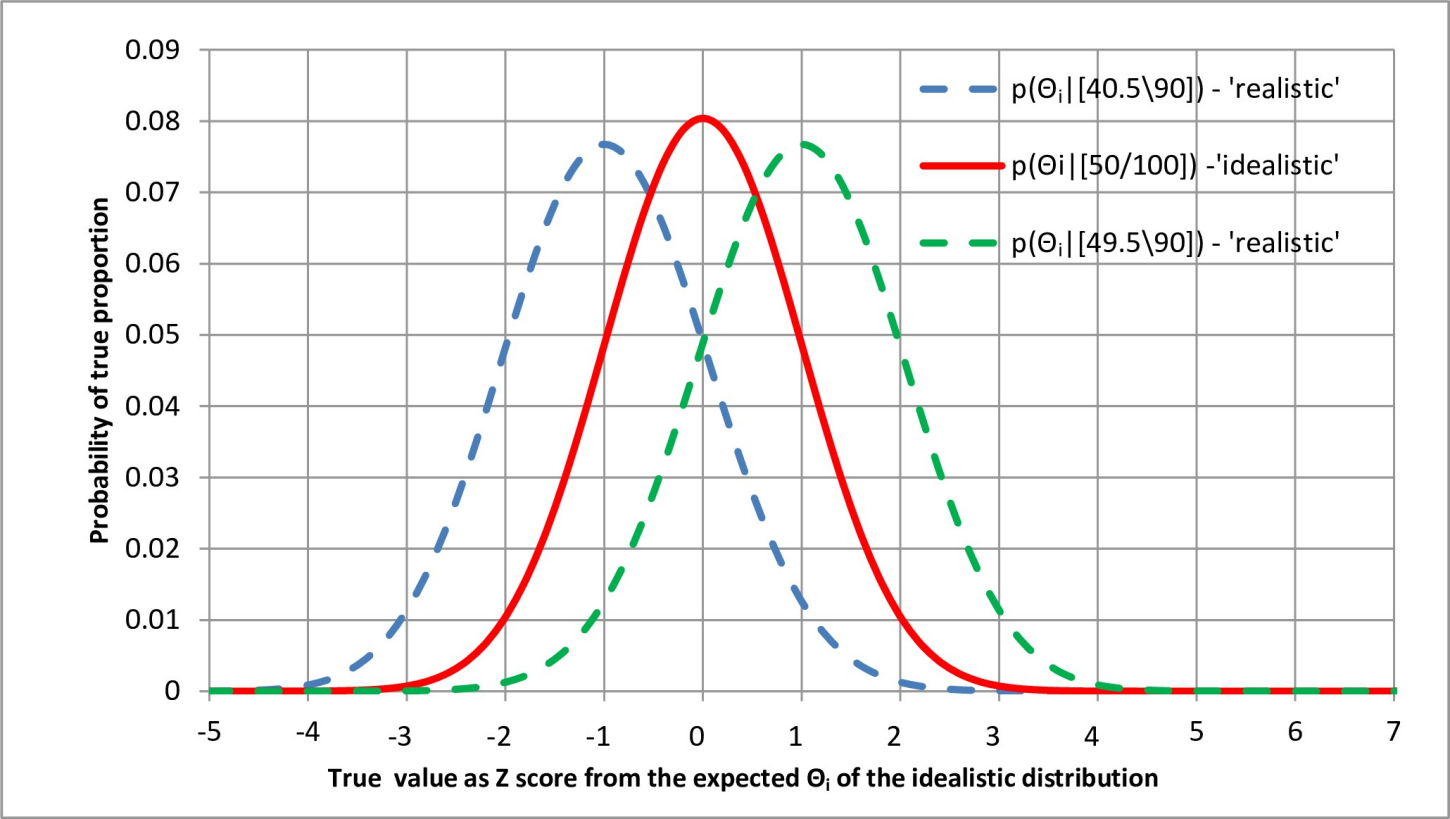
The effect of a probability of impeccable reproducibility on the probability of replication based on Z scores representing *Θ*_*i*_.

The middle of the three distributions in Fig 7 based on 100 observations is symmetrical. One hundred observations were used here instead of 99 to provide a symmetrical curve so that it could also be modelled with a Gaussian distribution with a standard error of σ = ((*Θ* * (1−*Θ*))/*n*)^1/2^ = ((0.5 * (1−0.5))/100)^1/2^ = 0.05. The likelihood distribution and posterior probability distribution can therefore be represented as in Fig 8 in terms of Z scores as baseline. However, a change in the reliable data from [50\100] to [45/90] would also affect the standard error, which would increase from 0.05 to ((0.45*(1–0.45))/90)^1/2^ = 0.05244. This represents a change of an extra +/- 0.00244 from the mean of each biased curve compared to the stand error of 0.05 for the central idealistic distribution. This is reflected by the slightly increased width of the realistic distributions on either side of the central realistic distributions in Figs 7 and 8. The effect of possible bias on the realistic probability of replication appears to be far greater than the effect of increased variance when allowances are made for unreliable observations.

It is apparent from Fig 8 that a probability of impeccable reproducibility (IR) conditional on an informal assessment of the Study Methods (SM) of *p(IR|SM)* = 0.9 results in a possible change of approximately +/- (1–0.9)*10 = +/- 1.0 standard error in the probability of replication and non-replication (based on bias and ignoring the small effect of variance). In other words, if the idealistic probability of non-replication was 0.025, corresponding to a Z score of -1.96, then the realistic Z score based on a *p(IR|SM)* = 0.9 would be from -1.96–1 = -2.96 to -1.96 +1 = -0.96, giving a probability of non-replication between 0.0015 or 0.1685. In practice, the probability of replication is unlikely to be greater when the method is poorly reproducible. The realistic probability of non-replication of greater than 39.2% in Fig 7 would therefore be represented by a range from the realistically pessimistic lower estimate of 1–0.1685 to the idealistic 1–0.025 (i.e. a probability of replication from 0.8135 to 0.975).

The suggestion of using a P-value of 0.005 (or a one-sided P-value of 0.0025) based on a Z score of -2.81 as a level of significance instead of a one-sided P-value of 0.025 [15] is of interest in this context. The level of significance of 0.0025 would correspond to a probability of idealistic replication of 0.9975 of a true outcome less severe than the null hypothesis. In order to get a realistic probability of replication of 0.975 less extreme than the null hypothesis, the expected Z score difference for *p(IR|SM)* would be 2.81–1.96 = 0.847, suggesting therefore that an approximate *p(IR|SM)* of 1–0.0847 = 0.915 is being applied. However, if the *p(IR|SM)* were 1 so that the realistic and idealistic probabilities of replication were the same, the Z score of 0.847 would also represent a probability of 0.8015 of getting a one-sided P value of 0.025 or less (or subject to assumptions of symmetry of distributions, replicating a result giving a probability of replication of 0.975 or more). This of course corresponds to the original study having a power of 0.8015 of achieving a one-sided P value of 0.025 if the study were repeated in exactly the same way with the same number of observations.

It should be noted that Fig 8 can also be applied to the results of any analysis based on a Gaussian model, whether it models continuous variables or proportions. The calculation is simple. If the *p(IR|SM)* is *x* (e.g. 0.9) and the idealistic Z score is y (e.g. 3.0), then the realistic Z score will be approximately

y+/−{(1−x)*10}[e.g.3+{(1−0.9)*10}=4;OR3−{(1−0.9)*10}=2].

In the absence of an informally estimated *p(IR|SM)*, it would be safer to regard the idealistic probability of replication (e.g. of 0.9975) as an upper bound of the realistic probability of replication (e.g. 0.975). Others have suggested previously that a one-sided P-value could be regarded as a lower bound for the corresponding posterior probability (i.e. of non-replication) [4, 5].

## 4. Discussion

### 4.1. Choosing a replication range

The examples have often used the null hypothesis to form one limit of a replication range. The probability that the true outcome lies within any range (e.g. beyond the null hypothesis or between 40% and 60% or above 40% etc.) is calculated by adding all the discrete probabilities within that range. (In the case of continuous distributions, this is achieved by normalising the likelihood probability densities and then integrating them.) The range can have an upper and lower bound (e.g. 40% to 60%) in an analogous way to a credibility or confidence interval. They could be chosen by specifying 95% posterior probability ranges with a probability of 0.025 that a true interval would fall into each tail. These ranges would summarise the data and provide an idealistic probability of replication, which means that all observations in an initial study were assumed to be made with impeccable accuracy and consistency and any subsequent studies were also done in an identical way to the original study so that they could be modelled by the mathematics of random selection. The range could be adjusted so that there was an idealistic posterior probability of 0.95 or 0.99 (for example) of a study being replicated within that range. It would be analogous to a 95% or 99% credibility or confidence interval.

### 4.2. Testing diagnostic and scientific hypotheses

The term hypothesis can be applied to a particular true outcome (e.g. a proportion of 60%) or to a replication range of such outcomes (e.g. 50% to 70%) or a scientific hypothesis. The result of a study might support another hypothesis (e.g. that the treatment was truly efficacious). The definition of ‘efficacious’ would be agreed according to some convention determined by a true result falling within a specified replication range (e.g. a true response rate greater than that of a placebo). If the probability of the true result falling into this range were 0.975, then it follows that there would also be a probability of 0.975 that the hypothesis of being truly efficacious would be correct. In this situation there would only be two hypotheses: that the treatment was efficacious or not.

The possibility that a sample had been drawn from a particular sub-population source out of many as displayed in Fig 1 is an example of multiple hypotheses. If the prior probability distribution in Fig 1 were hypothetical, then this could be updated in a Bayesian manner by using a likelihood distribution to give a new hypothetical posterior Bayesian distribution. However, the probabilities of having selected a sample from a source population can also be calculated by using the idealistic or realistic posterior distribution in Fig 7. This is because the posterior odds are equal to the likelihood ratio when the posterior odds are calculated using uniform prior probabilities. Thus although Figs 7 and 8 display posterior distributions, their odds can also give the same result as likelihood ratios, which is why the odds can be used to update Bayesian priors. In addition to this, the odds of a result falling into a replication range conditional on an observed result will be equal to the likelihood ratio of the observed result conditional on the replication range and its complement. This property means that when the marginal prior probabilities are uniform, the odds can be used to update a frequentist or Bayesian prior probability during scientific hypothesis testing or differential diagnosis.

### 4.3. Competing scientific hypotheses

It is common to have a number of competing scientific hypotheses. For example there may be a number of hypotheses as to why a new treatment reduces the frequency of renal disease in a randomised controlled trial. There could be three explanations: (1) A lowering of systemic blood pressure (BP) in all the body’s blood vessels that also protects the kidney, (2) a lowering of systemic BP generally and also an extra lowering of BP within a part the kidney (3) some other mechanism not yet considered. A study could be carried out that compared an old treatment thought only to lower systemic BP with the new treatment that was also thought to lower BP within a part of the kidney. If the new treatment had a greater protective effect despite the lowering of BP being the same in each treatment limb, then this could by agreement (e.g. within a society of nephrologists), mean that hypothesis (1) of lowering the BP generally was not the sole underlying mechanism.

If the probability of replication by getting a true result on the new treatment giving better protection than the old treatment was 0.975, then the probability of hypothesis (1), a general BP lowering effect being the only explanation would be 0.025. However this would not mean that the probability was 0.975 that hypothesis (2) of additional BP lowering within the kidney was correct, because there could be another explanation (hypothesis 3) that had not yet been considered. The probability of 0.975 would be either that hypothesis 2 or 3 was correct. Further support for hypothesis 2 could be sought by thinking of other specific hypotheses and showing that they too were improbable compared to hypothesis 2 (in the way that hypothesis 1 was shown to be improbable). This might happen if a second study gave a result that could occur commonly in hypothesis 2 but rarely in the new hypothesis. Karl Popper called this severe testing and that it was also impossible to prove that a scientific hypothesis was correct as there was always a possibility that the true hypothesis had not been considered yet. Mayo has long emphasised the need to incorporate severe testing into statistical inference [16].

### 4.4. Comparisons with medical diagnosis

The reasoning process used in scientific hypothetico-deductive reasoning is very similar to that used in medical differential diagnosis and can be modelled using a probabilistic elimination theorem [17]. However, in the medical situation, the same diagnostic hypotheses are tested again and again in different patients and much experience can be built up about the frequency with which each diagnosis in a list is eventually confirmed and the frequency with which something not in the list of diagnoses is discovered during day to day care.

If a patient with diabetes mellitus has an albumin excretion rate (AER) of 10mcg/min on a single sample then the probability of this exceeding a true threshold of 20mcg/min would be very low. The probability of the patient having diabetic renal disease or any other condition that causes increased protein in the urine would therefore be very low also. The patient could be reassured that no action has to be taken except for doing another test at some point in the future. This type of negative result is what would be hoped for in a screening test. However, if the AER were 40mcg/min then the probability of the true AER being less than 20mcg/min is now very low and the probability of it being greater than 20mcg/min would be very high. This result gives rise to a number of differential diagnoses (or hypotheses) of which diabetic renal disease is one possibility. The latter would be confirmed by a convention of showing the presence of other test results that would be common in diabetic renal disease but rare in rival possibilities. For example, the absence of white cells or other markers in a urine sample would be very unlikely in someone with a urinary tract infection making the latter less probable but a common finding in someone with diabetic renal disease making the latter more probable. If all other explanations can be shown to be highly improbable then by convention a working diagnosis of diabetic renal disease would be confirmed. However, this is subject to an understanding that the true explanation might be some other condition not yet discovered. This Popperian reasoning is modelled by the theorem of probabilistic elimination [17] derived from the extended version of Bayes’ rule. The reliability of this reasoning depends on tests having as high a precision as possible.

The precision of the test is assessed by finding the mean and standard deviation of a number of repeat measurements that are assumed to be sampled from a single source with a single true value. Because the prior probabilities of possible true values can be assumed to be uniform as explained in section 2.1, the normalised posterior distribution allows an idealistic probability to be estimated that the true result will fall into a specified range.

### 4.5. The precision of test results and their relationship to precision medicine

Precision medicine is an ideal that involves making precise predictions about diagnoses and other outcomes with or without interventions. We often need to combine many observations in order to increase the probability of predictions being correct. For this we need to find better observations where there are greater differences in the likelihoods of such observations occurring in the different outcomes we wish to predict. When the observations are the numerical results of measurements we deal with likelihood distributions. We then have to try to increase the difference between the means of such distributions and minimise their spread. Dichotomous observations (e.g. genetic markers) may not be very predictive alone in medicine and usually have to be combined with measures of severity in order to predict benefit from interventions. Mild symptoms or equivocal test results will often resolve or stabilise due to the body’s self correcting mechanisms and not require intervention.

New tests with greater differences between likelihoods of occurrence in different outcomes may be discovered by reasoning from hypotheses based on various scientific disciplines of physiology, biochemistry, genetics etc. and combinations of these based on mathematical modelling [18]. A possible improvement may also be suggested by a chance finding perhaps aided through data mining of big data sets. Such a hypothesis would suggest that there may be a helpful difference between patients with and without an outcome or intervention when this is tested formally on a fresh set of data and by assessing the probability of replicating that formal study result. A part of this general approach is to exclude mimicking diagnoses that would blur the difference in distributions (e.g. excluding bladder infection from a group of patients with increased urinary protein during the assessment of treatment for a protein leak from the kidneys) [18].

The spread of distributions can be minimised by improving laboratory techniques (e.g. by minimising batch differenced in reagents) or using the means of a number of tests results. It can also be reduced by allowing for covariates in modelling calculations or by stratifying a group (e.g. into males and females). It can be achieved by using the same subject as a control in randomised controlled trials in cross-over studies. Another approach is to remove the effect of variation between patients by restricting a study to a single patient (e.g. during n of 1 trials) or establishing normal ranges for individuals before they become ill. These are examples of reducing variation from ‘between’ to ‘within’ patients. The latter is also described as ‘personalised medicine’. Another form of personalised precision medicine is the iterative process of adjusting doses of medication until the individual patient has responded in a precise way or to keep trying the next most probable diagnosis and treatment if the preceding most probable diagnosis or treatment has not been successful.

In all the above, the precision of medical practice can be raised by improving the precision of tests. The precision of scientific measurements is usually represented by the standard deviation of a number of repeat tests results made on the same source; the narrower the standard deviation the more precise the test. This summarises the likelihood distribution of the test’s results conditional on a true mean equal to the sample mean. However, as possible true results would be based on an equally large number of repeat measurements they would have equal prior probabilities as discussed in section 2.1. This makes it possible to calculate the posterior probability of the true result falling into any specified range. This posterior probability can also be used a measure of the test’s precision.

### 4.6. Choosing and applying diagnostic and decision thresholds

A diagnostic or treatment indication criterion is usually based on a test result threshold that can be arrived at by identifying the point at which the probability of some outcome becomes high enough to make a diagnosis or consider some clinical management decisions [18]. However, the imperfect precision of an individual patient’s single test result may produce an inconsistency. For example if the threshold for diagnosing diabetic albuminuria was an albumin excretion rate (AER) of 20mcg/min and an individual patient’s single test result was 21mcg/min, then the probability of the true result being above the threshold would be only just over 0.5. However, if the single result was an AER of 40mcg/min, then the probability of the diagnosis based on a true result being above the threshold would be very high. Similar thresholds can be set for scientific hypotheses and the probability of exceeding that threshold estimated in the same way by using the posterior probability of replication based on a uniform prior probability.

If the test result was being used to estimate the probability of another event (e.g. an AER result being used to predict future kidney failure) then this would be based on the prior probability of the event in the studied population and also the likelihood distribution of the test result in those with and without the event [18]. The precision of the posterior probability estimate would depend on three things: (i) the reliability of the estimate of the proportion with nephropathy in the studied population, (ii) the estimate of the means and variance of both distributions and (iii) the precision of the test used to derive the distributions and the particular result of an individual patient. By taking into account the variation due to (i), (ii) and (iii) above, it is possible to estimate the range of posterior probabilities of true values (e.g. a probability of 0.2 of terminal kidney disease with 95% credibility interval of 0.15 to 0.25) conditional on an individual patient’s test result.

If scientists have a clearer understanding of the probability of replication and the effect of measurement precision on the diagnostic and treatment selection process, then this could reduce the proportion of false findings signalled by Ioannides (2]. Such an improved insight might also increase the rate of new discoveries and result in them being applied more effectively to the diagnostic process and to shared decision making with the patient when choosing treatments on the basis of that patient’s particular symptoms and test results.

### 4.7. The instability of probability thresholds and statistical significant levels

If a study that had provided a probability of replication of 0.975 based on an observation of [50\100] was repeated a very large number of times, the subsequent mean of all the observed proportions would be about 50% but the proportions would therefore be less than 50% about half the time and more than 50% about the other half of the time in an analogous way to the effect of a test’s precision on the probability of a diagnosis as described in the previous section 4.6. However, if we regarded P = 0.025 (equivalent to an idealistic probability of replication of 0.975) as a critical cut-off point for statistical significance, then about 50% of repeat studies would not be statistically significant. This may on the face of it sound alarming, when in reality it is due to unremarkable noise. Goodman [19] has pointed out that this would be the case if we could assume equal prior probabilities of the long term outcomes of random sampling.

It has been shown here that it is not necessary to assume such equal prior probabilities; this is a special property by definition of random sampling for the purpose of replication. It may be helpful therefore to replace one-sided P-values (e.g. 0.025) with the corresponding idealistic probability of replication (e.g. 0.975). This might avoid the mistake of thinking that P greater than 0.025 was not significant. It would be clear that an idealistic probability of replication of 0.975 is hardly different from that of 0.974 even though 0.975 corresponds to the concept of being statistically significant and the 0.974 does not.

The idealistic probability of getting another one-sided P-value of ≤ 0.025 if a study were repeated with the same numbers conditional on the original P value of 0.025 is 0.5 is found by subtracting the Z value of the repeat original P value (-1.96) from that of the repeat P value (-1.96) to give a Z difference of zero, thus giving a probability of 0.5. The probability of doing so with an original P-value of 0.0025 and Z = -2.807 would be found by subtracting from the latter the Z value for P = 0.025 of -1.960 to give a Z value difference of -2.807-(-1.960) = -0.847. This would correspond to a Z value of +0.847 in order to estimate the probability of replication. The probability corresponding to the latter Z value of +0.8471 would then be 0.8015, which would also correspond to the power of 0.8015 of getting a P-value of 0.025 or less on repeating the study when the original P value was 0.0025.

### 4.8. The width of a replication range, statistical significance and planning new studies

Basing a replication range on a null hypothesis of no difference at all may be too flattering because of its width. For example, if we are comparing two treatments and wished to know if the new treatment was better than an old treatment that had a cure rate of 40%, then a null hypothesis of 40% response would include bare differences (e.g. of 40.5%) in the replication range. This difference of 40–40.5% = 0.5% represented by a new treatment response rate of 40% may not be helpful in practice. It would be up to the individual who wished to act on the information to decide how much better than the old treatment the new treatment should be. Instead of placing the cut-off point at 40% as in Fig 4 giving a probability of replication of 0.975, the decision-maker might choose 45% to provide a minimum difference in treatment response rate of 45.0–40 = 5%. The probability of replication within the difference range of over 45% then would be 0.819.

If we calculate the probability of getting the same P-value or the something more statistically significant when a study is repeated, then the replication range becomes much narrower. The idealistic probability of replication would thus be lower and the realistic probability of replication may be lower still. Thus if the probability of getting on repetition a one sided P-value of ≤ 0.025 conditional on an initial P-value of 0.025 were 0.5 and the p(IT|MA) were 0.9, then the realistic probability of replication would be ≥0.158 (i.e. the realistic Z score would be 0+/- (1–0.9)*10 = -1 or +1).

If the initial P-value were 0.0025 then the idealistic Z score would be -2.807-(-1.96) = -0.847. If the p(IT|MA) were 0.9,the realistic Z score for the probability of replication would be 0.847-(1–0.9)*10 = -0.153. This Z score would give a realistic probability of replication of a pessimistic ≥0.439 for a P ≤ 0.025 next time. These probabilities of replication are of the same order of magnitude as those found by the Open Science Collaboration [10]. However if the initial study gave a very impressive P-value of 0.000001 and p(IT|MA) were 0.9, then the idealistic replication Z score would be 4.753–1.959 = 2.793, the realistic Z score could be 2.79 +/- (1–0.9)*10 = 1.793 or 3.79, giving a realistic probability of replication of ≥0.9635.

It is therefore important to be clear about the replication range. When a new study is being planned so that if it were to be repeated by someone else in the same way and the second study should also achieve a one-sided P value ≤0.025 or an idealistic probability of simple replication of ≥ 0.975, then the calculation will be have to be based on the power of 0.8 of achieving one-sided P value of 0.0025 in the first study (i.e. equivalent to a previous imaginary pilot result with an SEM of 0.847 + 2.81 = 3.66 from the null hypothesis). This would be equivalent to a power of 0.955 of getting a one-sided P-value of 0.025 in the first study (0.955 corresponding to an SEM of 3.66–1.96 = 1.7 from the null hypothesis). Although this might be achieved by increasing the number of observations, this might not be justified if the expected effect size or difference between distribution means were so small and clinically insignificant that the predicting test or observation would not help by making patient management more precise.

### 4.9. The concept of failure to replicate

If a study ostensibly performed in an identical way to a previous study came up with a very different result, it might be said that the second study had failed to replicate the first one. However, this could only be said for sure if both studies were based on a very large or infinite number of samples and only the first result fell into its specified replication range. Failing this condition for replication based on an impossibly large number of observations, another convention has to be used based on the probability of replication. One way of doing this would be to pool the data from both studies and to estimate the probability of replication within that range calculated conditional on the result of the pooled data set.

If both data sets were true random samples from the same population then the probability of replication should increase from that calculated using the first study result. However, if the probability of replication fell, then this lower probability of replication could be taken as a sign that an investigation should be conducted into possible reasons for non-replication in the long term. This might involve trying to identify factors that would have prevented impeccable reproducibility in the first and second studies (or lowered the probability) and repeating the study by including these safeguards. This could result in one or both studies being allocated a low probability of impeccable reproducibility and lower realistic probability of replication in retrospect. The threshold for taking a decision to investigate could vary depending on the seriousness of the implications. Therefore, ‘failure to replicate’ would be a general term for any stage in an investigation that would summarise and explain a decision to perform an investigation into possible non-replication. It would be a working hypothesis to justify taking action in the same way that a working diagnosis justifies a provisional clinical management strategy.

### 4.10. Choosing prior probabilities

Senn has emphasised the variety of ways in which Bayesian prior probabilities are chosen for statistical and the dubious nature of some of these ways [20]. With this in mind, it is important to distinguish between the types of hypotheses under consideration. It has been shown here that in order for a Bayesian prior probability distribution to be used for replication, it should be based on postulated data that might have been obtained from the current study’s methods, perhaps based on experience of similar previous studies. This does not include prior probabilities of population proportions (e.g. the distribution of the prevalence of people with the genetic marker in various sub-populations does not affect the result of random sampling of a particular population being studied).

### 4.11. The advantages of assessing the probability of replicating study results

The advantages of using the probability of replication over the P-value and confidence intervals are summarised in Table 1.

**Table 1:** Some comparisons between probability of replication, P-values and confidence intervals.

Probability of replication	P-values and confidence intervals
More intuitive for non-statisticians and the concepts potentially easier to learn quickly	Counterintuitive and confusing for non-statisticians and concepts difficult to learn
May allow a better mutual understanding between statisticians and non-statisticians	Associated with long-standing frustration between statisticians and non-statisticians
Allows degrees of certainty in the reliability of study findings to be expressed in a nuanced way e.g. there is little difference between the probability of replication of 0.95 and 0.94	Encourages cut and dried views of significant and non-significant study findings even for small differences e.g. p = 0.05 is significant and P = 0.06 is not significant
Possible to adjust a calculated idealistic probability of replication by taking into account the quality of study methodology to give a realistic probability of replication	No clear way of taking methodological issues into account except contested Bayesian prior probabilities or making the significance level more severe e.g. shifting it from 0.05 to 0.005
Separates the probability of study result replication from the application of the result to test scientific hypotheses	The null hypothesis does not directly assess probability of replication but combines it with scientific hypothesis testing to give a P value
Allows study results to be used in a hypothetico-deductive manner by using severe testing to show that one or more out of a list of scientific hypotheses are improbable	Difficult to use for Popperian severe testing but exhortation that this should be done [16]
Allow analogies to be drawn with sophisticated hypothetico-deductive differential diagnostic reasoning using dichotomous and continuous variable test results	Allows analogies to be drawn only with very simple dichotomous screening test results assessed in terms of sensitivity and specificity, which have limited roles in diagnosis
Useable in a Bayesian manner to combine probabilities based on personal impressions with fresh data	No equivalent methods available
Can be combined with Bayesian estimates to do sensitivity analyses for future studies	No equivalent methods available
Useable in a frequentist manner to perform a meta-analysis using past and current data by combining their likelihood distributions	Equivalent methods available
Compatible with Bayesian and Frequentist concepts, which might be reconciled	P- values criticised by Bayesians but accepted by Frequentists who criticise Bayesianism
An approach often aspired to and may therefore attract goodwill. May help to resolve the replication crisis	A long history of controversy about these methods with no clear prospect of resolving the replication crisis

## 5. Conclusion

The central point of this paper is that during random selection for estimating the probability of replication in the long term, the marginal prior probabilities of all possible outcomes are uniform. This is because replication within a specified range is determined by performing a very large number of hypothetical samples to conduct an ideal study. The number of observations of a typical study can be regarded as an initial sample taken from this very large ideal sample. The hypothetical large samples corresponding to each possible outcome of such an ideal study would contain the same number of observations and would therefore be equally probable. The prior probabilities of different outcomes in some known or unknown source population is therefore immaterial to the calculation of a probability of replication.

Many implications of the principle of uniform prior probabilities have been discussed when calculating the probability of replication and some of these are summarised in Table 1. One informal way has been suggested of calculating a realistic probability of replication from an idealistic probability of replication by taking into account an informally estimated probability of impeccable reproducibility of the study methods. There may be better ways of doing this. The idealistic probability of replication can be regarded as the upper bound of the realistic probability of replication. The idealistic frequentist posterior probability of replication may be easier for non-statisticians to understand and to apply than the troubled concept of the P-value.
